# Study on the Lamb Meat Consumer Behavior in Brazil

**DOI:** 10.3390/foods10081713

**Published:** 2021-07-23

**Authors:** Heloísa Valarine Battagin, Begoña Panea, Marco Antonio Trindade

**Affiliations:** 1 Faculty of Animal Science and Food Engineering, University of São Paulo, Av. Duque de Caxias Norte, Pirassununga 13635-900, Brazil; trindadema@usp.br; 2 Production Unit and Animal Health, Centro de Investigación y Tecnología Agroalimentaria de Aragón (CITA), Avda. Montañana 930, 50059 Zaragoza, Spain; bpanea@cita-aragon.es; 3 Instituto Agroalimentario de Aragón—IA2, CITA-Universidad de Zaragoza, 50059 Zaragoza, Spain

**Keywords:** preferences, quality cues, attitudes, categorical principal component analysis

## Abstract

In Brazil, the sheep market, including lamb meat consumption, is regionalized, and the consumption of lamb meat is higher in production areas; yet, consumption of lamb still remains below that of other meat livestock. The aim of this study was to identify the profile of Brazilian lamb meat consumers in order to understand their behavior in relation to food in general and on the consumption of this species. Therefore, a survey on consumer habits and preferences regarding food buying and consumption habits, their preferences in relation to the quality attributes of lamb meat, and sociodemographic characterization was performed. Data collected were analyzed by nonlinear canonic relationship analysis and categorical principal component analysis, followed by multiple factor analysis. Three consumer profiles were identified: traditional, interested, and disinterested, and a fourth group was considered independent but could not be described. Among lamb meat consumers, men with higher income seemed to be more frequent consumers than the others, and the intrinsic characteristics of meat quality, especially color and freshness, were the most important at the time of purchase. Thus, the results could be important to the industry in order to guide marketing strategies to certain niche markets.

## 1. Introduction

Lamb meat is nutritionally very rich, as it contains proteins, fatty acids, vitamins, and minerals of great importance for the maintenance of vital human functions [1]. Its consumption varies widely in different regions of the world, and an increase can be observed in developing countries [2]. In Brazil, sheep farming is characterized by a regional concentration of the national herd; in general animals, are raised on family-based, small farms, playing a secondary role in the property, and informal market of products is commonly observed [3].

Despite challenges for the growth of the sheep-farming production chain, the latest agricultural censuses in Brazil indicate production growth, with the Brazilian herd representing 1.5% of the world herd, the largest in South America, concentrated in the northeastern and southern regions of the country [4]. These regions are recurrently influenced by extreme weather events and reduced rainfall, which represents an advantage of the sheep farming in relation to other species, such as cattle and poultry, due to the greater adaptability of sheep and lower need for inputs. In addition, sheep products have greater turnover since the animal production cycle is shorter [3]. On the other hand, in farms in the midwestern region, the combination of beef and sheep farming is a common practice, usually aimed at own consumption and sale of surplus for cultural reasons and because the predominant activity in the region is cattle raising [4]. The lack of structure in the sheep-farming production chain in Brazil is also a limiting factor for growth, but this scenario has changed mainly in the southern and northeastern regions, with collective marketing being performed by cooperatives [3,4]. In these places, in addition to production, sheep consumption culture is greater than in other regions, which contributes to the increase in the annual average consumption per inhabitant, which is around 0.6 kg [4]. Other species, such as cattle, swine, and poultry, are more popular and consumed in the country. The consumption of lamb meat is associated with commemorative dates, and it has been observed that the sheep industry faces difficulties in reaching consumers who are not yet familiar with the species in addition to limited data on the perception of this product [5].

These dissimilarities in the interest of consumers among meats of different species and regions can be attributed to numerous factors, such as traditional foods in locations where they are produced, availability, cost, family habits, product perception, sociodemographic characteristics, and lifestyle in relation to food [6]. Consumer behavior in relation to meat also depends on familiarity with products [7] as well as market and psychological aspects, and the meat industry can increase its competitiveness by providing more information about products and production methods [8]. Competitiveness is linked to the understanding of the relationship between products and consumers, and market segmentation is a requirement to understand this relationship [6].

Consumer segmentation was widely studied and applied to several issues related to consumption habits and food perception: food benefits [9], healthy eating [10], emotional associations to meals [11], food-lifestyle convenience [12], organic products [13], and meat consumption [14,15,16,17,18,19]. Some studies have been carried out in Brazil [20,21,22,23,24] but none related to lamb meat. Groups of lamb meat consumers were identified in Spain, and segmentation indicated profiles termed “gourmet”, “conservative”, and “disinterested” and “basic” [6] or “traditional, “uninvolved”, “adventurous”, and “careless” [25,26]. All these papers showed important references to guide industry to expand lamb meat marketing.

Therefore, with the objective of identifying the profiles of Brazilian lamb meat consumers, this work sought to better understand food buying and consumption habits and preferences of lamb meat consumers in relation to meat quality attributes, seeking information for the industry to have data to outline marketing strategies based on what is more important for consumers. Therefore, the research was based on six research questions:

Q1. Whether the purchase or consumption habits of food in general or lamb meat depend on whether the consumer lives in a rural or urban area.
Q2. Whether the purchase or consumption habits of food in general or the perception of lamb meat quality depend on the region of the country where the consumer lives.
Q3. Whether sociodemographic variables (age, gender, schooling, and income) affect the purchase or consumption habits in general or of lamb meat.
Q4. Whether the consumption habits or perception of lamb meat quality are related to the frequency of lamb meat consumption.
Q5. Whether the groups of consumers that will consume lamb meat can be predicted and consumer profiles identified.

## 2. Materials and Methods

An online survey was conducted with the Google Forms tool from Google, Inc. (Menlo Park, CA, USA) and applied between the months of May and June 2019. The territory that was desired to be reached with the research was restricted to Brazil, with the participation of the largest number of Brazilian meat consumers. The research was exempted from the obligation to present a signed free and informed consent form when it was evaluated by the Human Research Ethics Committee of the Faculty of Animal Sciences and Food Engineering of the University of São Paulo.

The methodological framework of the lifestyle related to food (FRL) [27] was used to associate values and behaviors of consumers, and the questionnaire was divided into 6 blocks, similarly to [6,25,28]: (A) lifestyle-related variables in relation to food preparation and consumption habits; (B) variables related to food buying habits; (C) meat-buying habits; (D) importance of quality factors intrinsic and extrinsic to lamb meat at the time of purchase; (E) reasons for not eating lamb meat; and (F) sociodemographic variables.

The questionnaire (Table 1) was elaborated so that two key questions would direct respondents to specific questions: people who marked false to “I do eat lamb meat” did not answer sections (C), (D), and (E) and were directed to section (F); respondents who selected “never” for the frequency of lamb meat consumption did not answer section (D) and were directed to (E). Thus, only lamb meat consumers answered the question about meat quality criteria (D).

In the questionnaire, there is an unusual measurement unit for other countries, the minimum wage, which is a frequent way of measuring income in Brazil. The minimum wage is a value established by the Brazilian federal government that is annually updated. It is the minimum amount that must be monthly paid to people who work 44 h a week. The minimum wage is usually the monthly income of people without specialization. Specialized professionals usually earn higher salaries stipulated by unions. At the time of the research, the minimum wage in Brazil was R$ 998.00, and the questionnaire asked the family’s monthly income considering this criterion.

The online questionnaire was applied by sharing the access link via email, the WhatsApp application, and the social network Facebook, with disclosure to all contacts of researchers involved and at least one Facebook group dedicated to students from public universities of each of the 26 Brazilian states. After 40 days online, 1477 responses were collected, and 1457 were considered complete. Respondents who did not consume meat were excluded and valid responses were 1401. Table 2 contains the study sample demographic characteristics against the Brazilian national averages [29].

Respondents were mostly people with higher education and higher social classes, who live in urban areas. In addition, the percentage of responses from women in the sample was greater than the percentage of women in the Brazilian population. The last official demographic census carried out in Brazil that took into account the distribution of the population in different regions occurred in 2010, so it does not exactly indicate the current distribution of the population. The Brazilian population has grown since then, so there are approximate values that indicate the northern and northeastern regions were underrepresented, while the southeastern region was overrepresented.

Statistical analysis was performed using XLStat 17.03 (Addinsoft, Barcelona, Spain), with Kruskal–Wallis tests for research questions Q1 to Q4, study of frequencies by chi-square tests, and analysis of multiple correspondences. As the sample size is large, small variations between observed frequencies and those expected in the Pearson’s chi-square test can generate high chi-square values. A chi-square test was used, and the corrected standardised residual between the observed and expected cases within each cell greater than |1.96| was considered.

The study of how socioeconomic variables affect consumer behavior was performed through the nonlinear canonical relationship analysis and by the categorical principal component analysis (CATPCA). The first one consists of procedures that aim to understand in the best possible way the variance in relationships between two sets of numerical variables in a space of few dimensions. CATPCA allows reducing an original set of variables to find a smaller set of uncorrelated components that represent most of the information found in original variables. These analyses were performed for the group of 1401 valid responses using variables that showed a significant relationship with each other.

Research question Q5 was studied using the decision tree method and multiple factor analysis (MFA) [30] of XLStat 17.03 (Addinsoft, Barcelona, Spain.)

## 3. Results and Discussion

### 3.1. Influence of the Place of Residence

The Kruskal–Wallis test, which is a non-parametric method for one-way ANOVA, reported that food buying and consumption habits are not different for residents of urban or rural areas. The frequency at which the population living in urban areas have meals outside their homes is slightly higher than in rural areas (CC = 0.064), and individuals living in rural areas eat more organic foods than those living in urban areas (CC = 0.091). Among respondents who consume lamb meat at least once a week, a greater proportion of people are from rural areas (CC = 0.086).

The small differences found between rural and urban populations, were considered irrelevant due to the contingency coefficient being very close to zero. It may have occurred due to availability, as there is greater ease for residents of urban areas to find restaurants, while for those living in rural areas, there is greater availability of organic food and even lamb meat directly from the producer. This is very common in Brazil, where most of the herd is spread over small properties that are often family-based, and informal marketing is frequent [3].

Furthermore, respondents from rural areas were underrepresented. The fact that the questionnaire was online was a limitation for providing answers from rural areas that sometimes do not have internet access. This underrepresentation may have been reflected in results. Respondents from urban areas were expected to be more interested in foreign and gourmet options, but this has not been verified. Thus, the research question Q1 is rejected, and for the study of the following questions, the variable addressed here is no longer considered, and these two groups are now treated as a single population since separation is of no practical use.

### 3.2. The Regionality Factor

With the grouping of Brazilian states into each of the five regions of the country (southern, southeastern, midwestern, northeastern, and northern), a few habits and preferences were dependent on the region where the respondent lives, like enjoying cooking, foreign food, celebrating with friends and family, whether everyone at home cooks, time spent cooking, having meals only in restaurants, reading labels, using the shopping list when shopping for food, being interested in trendy and gourmet foods, as well as always choosing organic options. However, at the individual state-level within specific regions, variation was minimal. Furthermore, states did not prove important in defining food purchasing and consumption behaviors, as results across states were not significantly different from one another.

**Figure 1:**
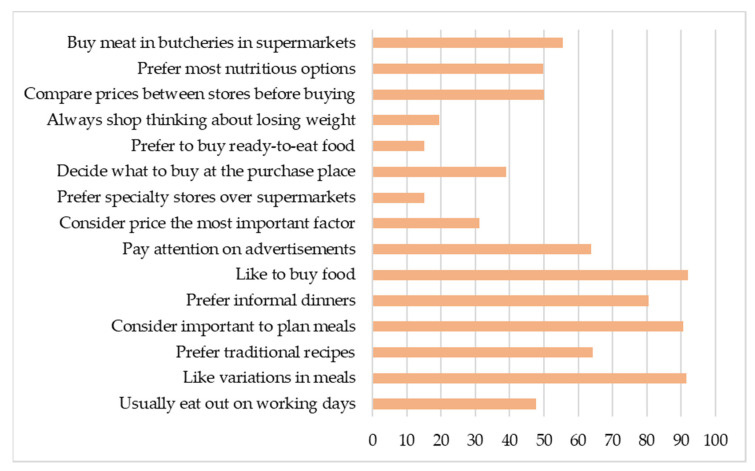
Food-buying and consumption habits and preferences that do not vary among Brazilian regions (percentages of the population).

In addition, the region of origin did not affect the distribution of responses regarding most food purchase and consumption habits covered in the survey (Figure 1). Thus, it could be inferred that the sample has the same behaviors in any of the Brazilian regions.

Considering the specific case of the buying and consumption habits of lamb meat, region and state do influence the proportion of people who consume lamb meat. The northeastern and southern regions presented higher consumption of this species than the other regions. As these are traditionally sheep producers and traders, consumption in these regions is also higher, but the northeastern region is the region with the highest growth in the consumption of this species [3].

In the southeastern region, which is formed by four states, São Paulo and Minas Gerais are meat producers. In Minas Gerais, there is a large number of small producers with an extensive production system. In São Paulo, the production system is predominantly intensive, focusing on commercial breeding of animals with higher added value and sheep for special cuts. It was observed that in the state of São Paulo, the most populous, more people consume lamb meat and also do so more frequently than in the other states in the region. This may be due to the greater purchasing power of residents of this state in relation to others since São Paulo is the richest state. On the other hand, these results may have been influenced by the sample, as the population of this region was overrepresented. In the midwestern region, the state of Mato Grosso do Sul is the greatest lamb meat consumer, and in the southern region, the largest consumption was observed for the state of Santa Catarina. Although Rio Grande do Sul is the largest sheep meat producer in the southern region, Santa Catarina has the largest number of establishments regularly operating for slaughter processes, with organized structure for meat production. The greater consumption in this state may be the result of this consolidated structure for product marketing.

**Figure 2:**
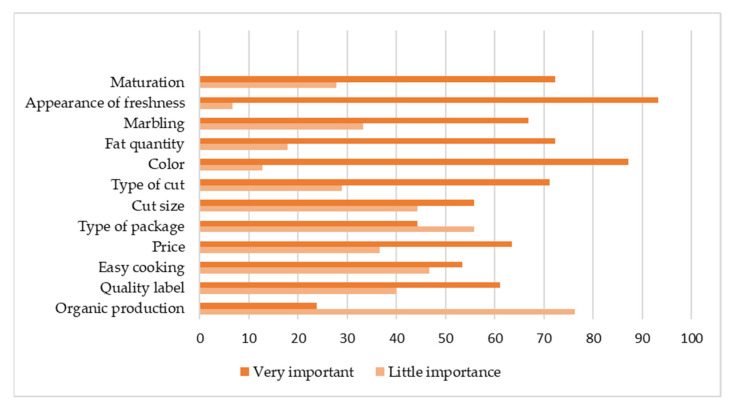
Distribution of the degree of importance that the sample attributes to the different quality criteria of lamb meat (percentages of the population).

Despite all variations described according to the different Brazilian regions, the analysis of multiple correspondences found that the origin of consumers did not lead to differences in the degree of importance that they attribute to intrinsic and extrinsic criteria of lamb meat at the time of purchase, according to the four-point scale of questionnaire section (D). These responses were then divided into only two groups (Figure 2): little importance (“very little importance” and “little importance”) and very important (“important” and “very important” answers).

At the time of purchase, survey respondents placed great importance on freshness (93.3% of responses) and meat color (87.2%), far beyond the importance attributed to other quality criteria covered by this study, as also found in other studies [26,31]. On the other hand, it was observed that organic lamb meat production is not an important requirement for 76.2% of respondents, as verified by [25], and for practical purposes, there is no demand for organic lamb meat in Brazil, and therefore, there is no need for investment in this regard by producers today. The type of packaging also showed to be unimportant for most respondents. Thus, the greatest concerns and investments of producers and industry must be focused on intrinsic criteria to attract those who are already lamb meat consumers, taking care that meat arrives fresh at points of sale.

As for the importance that consumers give to these criteria, these individuals need practical experience and knowledge to assess the quality of a product [26]; therefore, it is expected that the quality attributes assessed at the time of purchase were more influenced by the other sociodemographic variables than people’s origin.

The analysis of multiple correspondences also indicated that there is no relationship between intrinsic and extrinsic characteristics under study. Thus, research question Q2 can be rejected.

### 3.3. Consumer Habits and Sociodemographic Characterization

Considering that living in urban or rural areas or in a certain region of the country did not influence habits and perception of lamb meat quality, the sample was treated as a unique population to analyze whether or not there is influence of sociodemographic characteristics.

Age, schooling, and income affected 80% of the food buying and consumption habits under study and even whether people are lamb meat consumers or not and their frequency of consumption. The frequency of lamb meat consumption was also indicated by [5] as the factor that most affected the perception of the Brazilian lamb meat consumer by the word association method. Among the 25 habits and preferences surveyed in sections (A) and (B) of the questionnaire, only five were not affected by any of these variables: variations in meals, planning meals, celebrating with friends and family, having meals only in restaurants, and trying everything that is trendy.

With a focus on the quality criteria that lamb consumers consider at the time of purchase, it is clear that the criteria that are strongly influenced are ease of preparation and price.

At the time of purchase, women were more concerned about the ease of preparation than men. As gender also influences the time spent cooking, and women cook more than men, it is possible that women give more importance to this aspect; while men also purchase lamb meat but in a much lower proportion, women will prepare it.

Price, in turn, was influenced by age and family income. Price is considered less important for people aged 45 or older than for younger people, while for people with monthly income of two to three times the minimum wage, this factor is more important at the time of purchase than for families with income higher than 10 times the minimum wage. Obviously, individuals with lower incomes need to be more careful when choosing foods precisely because they have less money available, and price is considered an important factor in the decision-making process, as found in other studies [6,25,26]. Price was considered less important for individuals in the most frequent consumption group, a result similar to that found by [5] in the word association test in which price was frequently mentioned by occasional lamb meat consumers but less frequently by those who consume lamb meat more frequently.

By grouping the quality criteria into only two groups (low and high importance), it was found that other criteria were affected so that the perception of the importance of meat certification is affected by age and gender, and the importance given to meat color is influenced by gender. Consumers up to 30 years of age and women place more importance on certification, and women care more about color than men.

The most relevant finding was that all sociodemographic variables influence whether people are lamb meat consumers and its frequency of consumption. Thus, it is possible that the fact of being a lamb meat consumer or not as well as the frequency of consumption are related to the consumer’s habits and preferences and to the meat quality criteria that they consider most important at the time of purchase. Therefore, research question Q3 was accepted.

### 3.4. Food-Consumption Habits and Perception of Lamb Meat Quality in Relation to the Frequency of Consumption

The Kruskal–Wallis test showed that lamb meat consumers have a positive relationship with preferences for foreign food, gourmet options, and specialized meat stores than non-lamb meat consumers and are less likely to use a shopping list when buying food. Analyzing lamb meat consumers separately, behaviors also vary according to the frequency of consumption of this food. Regarding the intrinsic and extrinsic criteria of lamb meat quality, the frequency of consumption revealed to be related to the importance given to cut and price, which are less important for the group that consumes lamb meat weekly than for the other groups.

**Figure 3:**
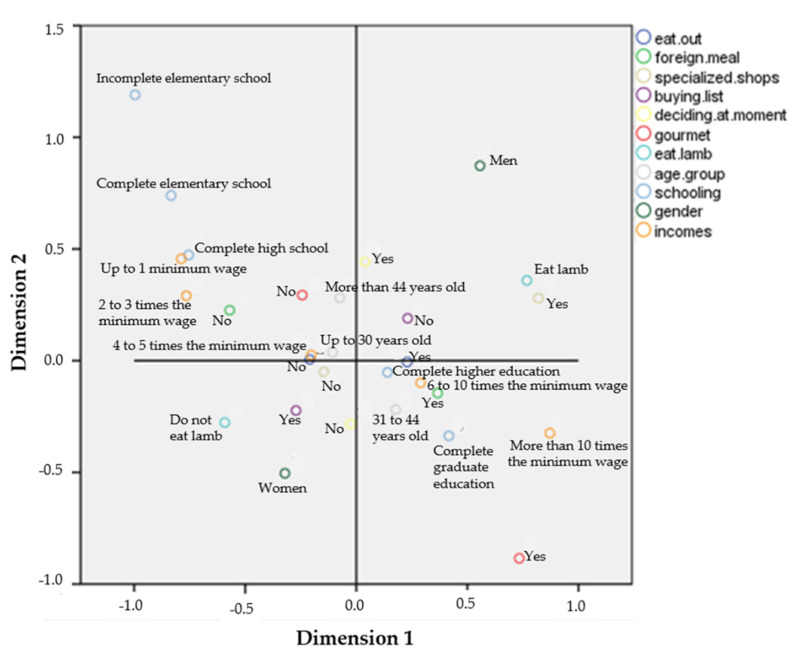
Centroids of all variables studied by nonlinear canonical relationship analysis.

Nonlinear canonical relationship analysis (Figure 3) indicated the three lowest income groups (up to five times the minimum wage) and the least educated people (completed high school at most) are those who do not have the habit of buying meat at specialized stores, do not usually choose gourmet options, and are not so interested in foreign food. In the opposite quadrant, the two highest income levels (from six times the minimum wage) and schooling levels (people with at least higher education) are more interested in gourmet options and foreign food. Among lamb meat consumers, those of higher social class consume more lamb meat and care more about marbling and cutting criteria, while for the group with income of up to three times the minimum wage, these criteria are of little importance. Younger consumers, up to 30 years old, care more about price, which is of little importance for older consumers and those with higher income. Regarding gender, men eat more lamb meat and generally decides what to buy at the time of purchase, while women are more used to using shopping list.

**Figure 4:**
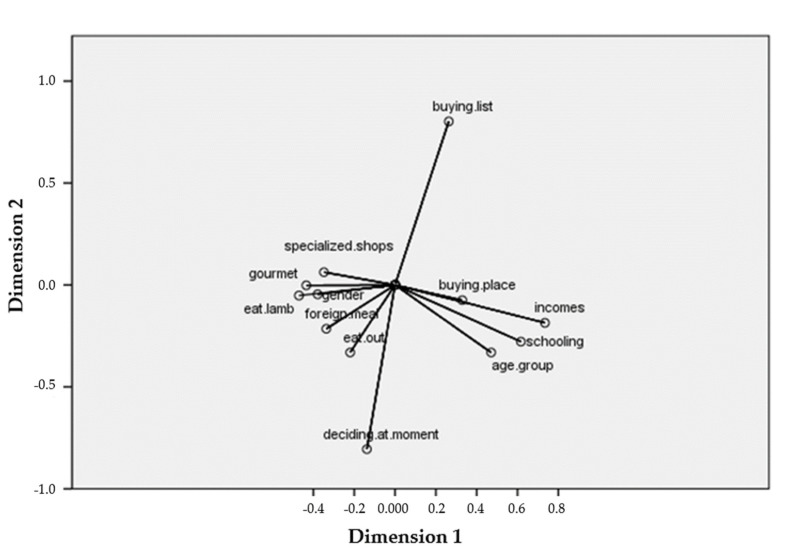
Graph of component saturations generated by CATPCA analysis.

The finding that women are associated with lower lamb meat consumption is recurrent [14,32,33], but CATPCA (Figure 4) indicated that gender has less influence than age, income, and schooling on behaviors presented by these individuals.

Thus, it was confirmed that research question Q4 was accepted.

### 3.5. Lamb Meat Consumption Groups and Consumer Clustering

**Figure 5:**
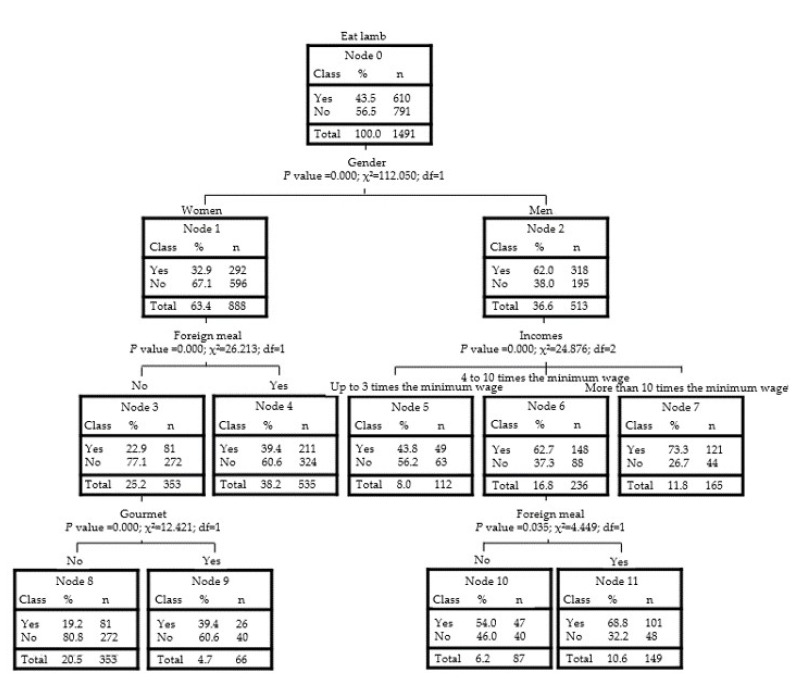
Decision tree of lamb meat consumption groups (growth method: exhaustive CHAID; dependent variable: lamb meat consumers).

Decision tree analysis (Figure 5), using only habits that in previous analyses were important, allowed verifying which group of consumers is more predisposed to consuming lamb meat. Classes were divided inside the boxes between “Yes” and “No” for people who eat lamb meat or people who do not, respectively.

When dividing the sample by gender, men are always more interested in consuming lamb meat. Among women, those who are more predisposed to consuming lamb meat are those who like foreign food and those who buy gourmet options, and in both cases, only 39.4% of women would consume lamb meat. On the other hand, men are more interested than women in any of the tree nodes. Among men, the higher the income, the greater the interest in consuming lamb meat (node 7), with the group of men with higher incomes being the group most likely (73.3%) to consume this meat.

Regarding the difference in the probabilities of these groups of men and women of consuming lamb meat, there is need to understand the motivations of women for not consuming it. To investigate this result, questions in section (E) were studied by frequency, and 23.2% of women who do not consume lamb meat claim to dislike this meat, 26.7% affirm that they do not consume it because they have never tried it, 9.9% consider it difficult to find, and 6.4% do not know how or find it difficult to prepare.

As for men who do not consume lamb meat, 25.1% do not like it, 13.6% have never tried it, 17.9% consider it difficult to find, and 5.1% do not know how or find it difficult to prepare. The other answers were related to factors such as lack of family habit, price, disgust, health issues, low availability of this type of product, and concern for animal welfare but in irrelevant proportions. For both genders, the main reason for not consuming lamb meat is not enjoying it, and this is a group of individuals that the industry cannot easily reach to increase consumption. The other most frequent reasons indicated by respondents do not coincide with what has been found in other studies, which address factors more related to the reduction of red meat consumption in general, mainly related to health issues [26,34].

However, these reasons informed by respondents can be circumvented by the industry, rural producers, and stores with the use of marketing tools to stimulate consumption by these individuals. Points in specialized meat stores, supermarkets, and butcher shops for customers to try the product near shelves where products are displayed for sale, promotions, and preparation suggestions on the packaging can be ways to attract this population. Manners of strengthening relationships and improving the market are ways to make it easier for the consumer to have information about the product [8] and be familiar with it [7,35].

**Figure 6:**
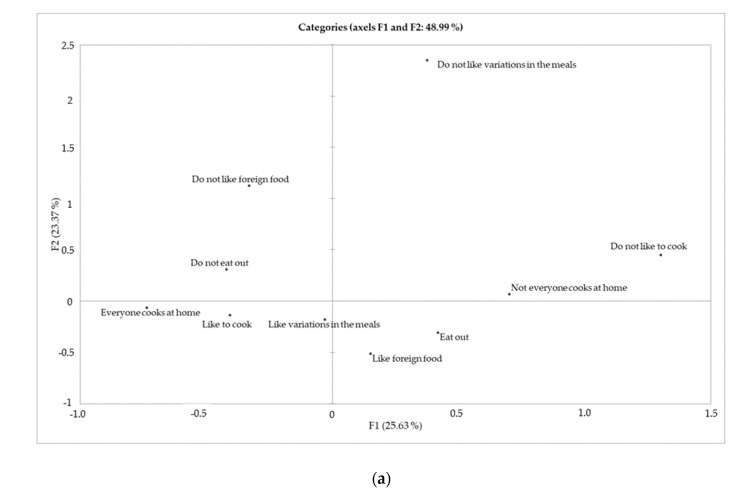

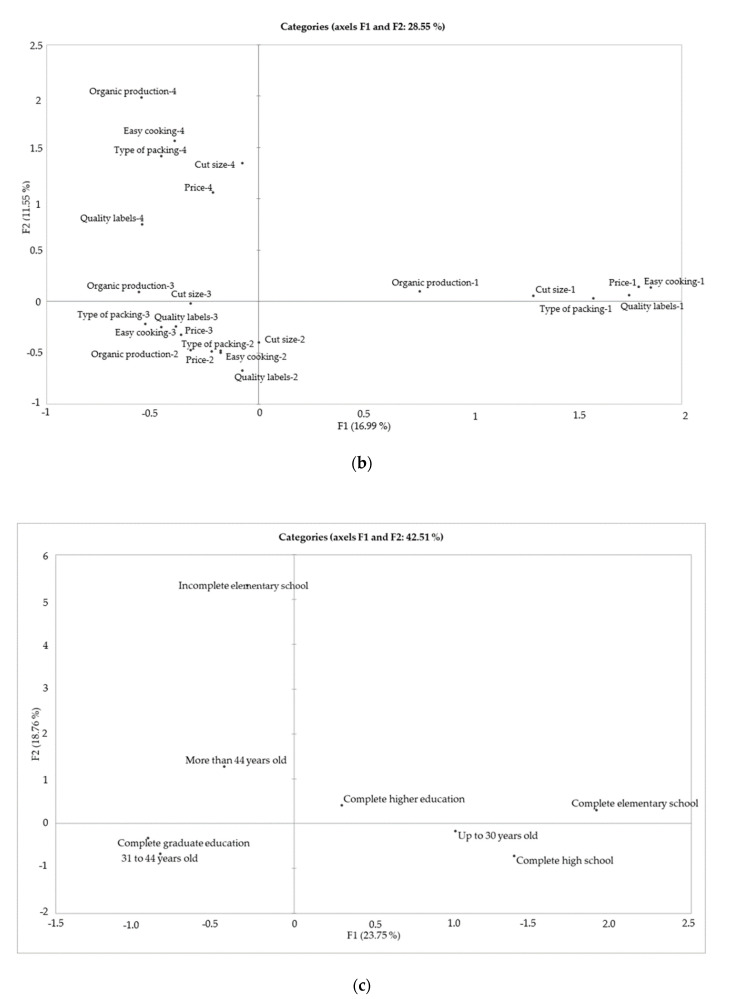
Consumer clustering by MFA: (**a**) Map of consumption habits and food purchases; (**b**) map of quality criteria assessed at the time of lamb meat purchase (1: Very little importance; 2: little importance; 3: Important; 4: Very important); (**c**) map of sociodemographic variables.

It is known that the segmentation of consumers based on their behavior in relation to food is significant for the formulation of marketing strategies for specific products because it informs the consumer’s particular choices [9]; therefore, multiple factor analysis (MFA) was performed for food purchase and consumption habits (Figure 6a), for quality criteria (Figure 6b), and for sociodemographic variables (Figure 6c).

Considering a juxtaposition of the three maps, the upper left quadrant includes people who considered meat quality criteria extremely important and who are mostly from the age group of 45 years, with minimum schooling (incomplete elementary school), who do not like foreign foods and who normally do not eat out on workdays. This consumer profile can be called traditional.

The upper right quadrant includes people for whom quality criteria are of little importance, who do not like to cook and to have variations in their meals, and live in homes where not everyone cooks. This group has predominance of people with complete graduation and complete elementary education, but there is no predominant age group. These people have a consumption profile called disinterested.

The lower left quadrant is composed of a combination of people who attribute intermediate importance to quality criteria (central regions of the four-point scale). They have complete higher education, are in the age group of 31–44 years, and like to cook, to vary their meals, and live in homes where everyone cooks. They can be called interested.

Finally, the lower right quadrant is composed of individuals who like foreign food, usually eat outside their homes, and who are younger (up to 30 years of age), with complete high school (possibly attending higher education). However, there are no data on the importance that this group attributes to the quality criteria of lamb meat at the time of purchase.

As there is information to describe the consumption profiles of only part of the interviewees, the groups of individuals who were separated into clusters were identified; however, it was not possible to describe them completely or to know the number of individuals in each cluster, which was a study limitation. Nevertheless, the consumer profiles of traditional, disinterested, and interested provide rich information for building targeted marketing strategies since this information is accessible to the industry and allows making the product more accessible to consumers. Therefore, research question Q5 was accepted.

## 4. Conclusions

In Brazil, age, schooling, and income affect food buying and consumption habits in general, but these behaviors are not influenced by the region of the country where the consumer lives. On the other hand, consumption habits and the perception of lamb meat quality depend on the frequency of consumption of this meat as well as on the sociodemographic characteristics of consumers. Parts of population with lower education and income were underrepresented, and these limitations should avoid in future studies using additional collecting methods, such as questionnaires with interviewers in person, in markets, and fairs, for example.

It is possible to predict which groups of consumers will consume lamb meat based on the study variables; however, the segmentation of these individuals into clusters was not completely possible due to data complexity, with categorical variables of different natures and sizes. Even so, men with higher income seem to be more frequent consumers than the others, and knowing consumer profiles of traditional, disinterested, and interested allows the industry to focus different marketing strategies in determined niche markets. In addition, the results obtained on the reasons of non-lamb meat consumers were relevant to understand the low consumption of this species in Brazil. It generated valuable information that can be used by rural producers, industry, and points of sales in order to outline marketing strategies for the growth of the sheep meat market, with focus on intrinsic quality criteria and promotion of more direct contact between product and consumer.

## Figures and Tables

**Table 1:** Questionnaire on food buying, preparation and consumption habits, meat-buying habits, and the importance of attributes intrinsic and extrinsic to lamb meat.

(A) Food Preparation and Consumption Habits								
We would like to know your food preparation and consumption habits. Please, mark true or false.								
Habit						True		False
I like to cook								
I usually eat out on working days								
I like foreign food								
I like variations in my meals								
In my home, everyone cooks								
Traditional recipes are better								
I spend a lot of time cooking								
Planning meals is important for family health								
I like to celebrate with friends and family								
My family prefers informal dinners								
I only eat in restaurants								
I eat lamb meat								
**(B) Food Buying Habits**								
We would like to know your food buying habits. Please, mark true or false.								
Habit						True		False
I like to buy food								
I pay attention to advertisements								
Reading labels is very important to me								
Price is the most important thing for me								
I prefer specialty stores over supermarkets								
I always follow a shopping list								
I decide what to buy when I arrive at the place of purchase								
I usually try everything that is trendy								
I prefer to buy ready-to-eat food								
I always shop thinking about losing weight								
I compare prices between stores/supermarkets before buying								
I always choose the most nutritious options								
I usually buy gourmet options								
I always opt for organic versions								
**(C) Meat Buying Habits**								
Where do you buy meat most often? ( ) Directly from the producer ( ) Traditional butcheries ( ) Butcheries in supermarkets ( ) Packaged (in supermarkets) ( ) Others								
How often do you eat lamb meat? ( ) 1 time or more per week ( ) 2 to 3 times per month ( ) 1 time per month ( ) Less than 1 time per month ( ) Never								
**(D) Importance of Attributes Intrinsic and Extrinsic to Lamb Meat at the Time of Purchase ^1^**								
How important do you consider the following extrinsic quality criteria of lamb meat?								
Attribute		Very little important	Little important		Important		Very important	
Organic production								
Quality labels								
Easy-cooking								
Price								
Type of packing								
How important do you consider the following intrinsic quality criteria of lamb meat?								
Attribute	Very little important		Little important	Important			Very important	
Type of cut								
Cut size								
Color								
Fat quantity								
Marbling								
Appearance of freshness								
Maturation								
**(E) Reason for not Eating Lamb Meat ^2^**								
Why do you not eat lamb meat? ( ) I do not like lamb meat ( ) I never tasted lamb meat ( ) My family does not like lamb meat ( ) Lamb meat is expensive ( ) Lamb meat is hard to find to buy ( ) I do not know how to prepare lamb meat ( ) It is hard to prepare lamb meat ( ) Other (please specify) ____________________								
**(F) Sociodemographic Characterization**								
Now, some data about you.								
Age ( ) Up to 30 years old ( ) 31–44 years old ( ) 45 years old or more								
Gender ( ) Female ( ) Male								
Schooling ( ) Incomplete elementary school ( ) Complete elementary school ( ) Complete high school ( ) Complete higher education ( ) Complete graduate education								
State of origin (Specify the state among the 26 Brazilian states)								
Family income ^3^ ( ) Up to 1 minimum wage ( ) 2 to 3 times the minimum wage ( ) 4 to 5 times the minimum wage ( ) 6 to 10 times the minimum wage ( ) More than 10 times the minimum wage								
Place of residence ( ) Rural ( ) Urban								

^1^ Only lamb meat consumers were directed to this question; ^2^ only non-lamb meat consumers answered this question; ^3^ considering the minimum wage of 1000 reais (approximately 200 dollars).

**Table 2:** Brazilian population and sample characterization by age, gender, place of residence, and schooling.

Age		
	National average	Sample
Up to 30 years old	42.3	43.3
31 to 44 years old	22.3	35.1
45 years old or more	35.4	21.6
**Gender**		
	National average	Sample
Female	51.8	63.4
Male	48.2	36.6
**Place of Residence**		
	National average	Sample
Rural	15.3	4.7
Urban	84.7	95.3
**Schooling ^1^**		
	National average	Sample
Incomplete elementary school	38.6	1.3
Complete elementary school	12.5	1.7
Complete high school	31.8	22.6
Complete higher or graduate education	17.4	74.5

^1^ For this parameter, the census considered people aged 25 or over.
